# Nutritional interventions to support broiler chickens during *Eimeria* infection

**DOI:** 10.1016/j.psj.2022.101853

**Published:** 2022-03-11

**Authors:** R.R. Santos, F.C. Velkers, J.C.M. Vernooij, L. Star, J.L.T. Heerkens, J. van Harn, I.C. de Jong

**Affiliations:** ⁎ Schothorst Feed Research, 8200 AM, Lelystad, The Netherlands; † Utrecht University, Faculty of Veterinary Medicine, Department Population Health Sciences, 3508 TD, Utrecht, The Netherlands; ‡ Aeres University of Applied Sciences Dronten, 8250 AJ, Dronten, The Netherlands; § Wageningen Livestock Research, 6700 AH, Wageningen, The Netherlands

**Keywords:** coccidiosis, nutrition, performance, broiler

## Abstract

Different combinations of gut health-promoting dietary interventions were tested to support broilers during different stages of *Eimeria* infection. One-day-old male Ross 308 broilers (n = 720) were randomly assigned to one of 6 dietary treatments, with 6 pens per treatment and 20 birds per pen, for 35 d. At 7 d of age (**d**7), all birds were inoculated with 1000, 100, and 500 sporulated oocysts of *E. acervulina, E. maxima*, and *E. tenella*, respectively. A 4-phase feeding schedule was provided. The dietary treatments (**TRT**) 1 to 4 included the basal diet supplemented with multispecies probiotics from d0 to 9 and coated butyrate and threonine from d28 to 35 but received four different combinations of prebiotics and phytochemicals from d9 to 18 and d18 to 28. The basal diet for the positive control (**PC**, TRT5) included diclazuril as a anticoccidial. The negative control (**NC**, TRT6) contained no anticoccidial. Performance was assessed for each feeding phase, and oocyst output, *Eimeria* lesion scores, cecal weight, litter quality, and footpad lesions were assessed at d14, d22, d28, and d35. Body weight gain (**BWG**) and feed intake (**FI**) were not affected by dietary treatment. PC broilers had the best feed conversion ratio (**FCR**) of all treatments from d0 to 35 (*P <* 0.001). None of the dietary treatments resulted in better litter quality or reduced footpad lesions compared to the PC. Moreover, the PC was most effective in reducing oocyst output and lesion scores compared to all other treatments. However, broilers that received the multispecies probiotics (d0 to 9), saponins (d9 to 18), saponins, artemisin, and curcumin (d18 to 28), and coated butyrate and threonine (d28 to 35) had the best FCR (*P <* 0.001) and lowest oocyst output and lesion scores compared to other dietary treatments. This study suggests that although the tested compounds did not perform as well as the anticoccidial, when applied in the proper feeding period, they may support bird resilience during coccidiosis infection.

## INTRODUCTION

Coccidiosis is an important gut health problem in poultry, caused by infections with a very contagious protozoan intestinal parasite (*Eimeria* spp.). *Eimeria* infections can cause malabsorption and enteritis, consequently reducing performance (higher feed conversion ratio and reduced growth rate), impairing litter quality and welfare (more contact dermatitis), increasing the risk of carriage of foodborne zoonotic pathogens and carcass condemnations at the slaughterhouse, and in severe cases, increasing mortality (Blake et al., 2020, 2021).

Coccidiosis has a substantial negative economic impact, mostly as a result of reduced production performance and costs for prophylaxis and treatment, with annual global costs for poultry production higher than €11 billion (Blake et al., 2020). Prophylaxis includes the preventive use of anticoccidial drugs, that do not completely prevent *Eimeria* replication in the gut (Noacket al., 2019). The widespread increase in occurrence of anticoccidial drug resistance and societal pressure to reduce the use of antimicrobials from a public health perspective (Agunos et al., 2017) has increased the search for alternatives, such as vaccination, botanicals, organic acids, immunomodulators, carbohydrates, probiotics, and prebiotics (Blake et al., 2021). In Europe, in contrast to the USA, the use of live anticoccidial vaccines, especially in broilers, has remained limited for four reasons: (1) Only attenuated anticoccidial vaccines are approved and their cost of production is significantly higher than that of nonattenuated vaccines, (2) The use of anticoccidials belonging to the polyether ionophore class is permitted in antibiotic-free production systems, (3) Consistent application may be challenging for farmers, and (4) Slower development of immunity than with non-attenuated anticoccidial vaccines (Blake and Tomley, 2014; Noack et al., 2019; Blake et al., 2020; Attree et al., 2021). Nevertheless, programs where live vaccines are alternated between production cycles with anticoccidials are increasingly being applied to restore sensitivity to anticoccidials (Kadykalo et al., 2018; Blake et al., 2021).

Although management and biosecurity measures can contribute to coccidiosis control, *Eimeria* infections cannot be completely prevented (Peek and Landman, 2011). Therefore, it is important to improve host resilience and reduce the negative impact of *Eimeria* and secondary infections on broiler welfare and performance. Nutritional interventions may improve gut health and therefore potentially reduce the negative impact of coccidiosis on broiler health and performance.

It is unlikely that single alternative ingredients other than anticoccidials will have the ability to cure or counteract severe *Eimeria* infections. However, Adhikari et al. (2020) suggested that combinations of ingredients may aid in early prevention, providing immediate control of intestinal damage and significantly reducing the impact of coccidiosis. For optimal resistance before, during, and after *Eimeria* infection: (1) intestinal development and an optimal balance of intestinal microbiota must be stimulated (Calik et al., 2019); (2) general host resistance and immune development must be supported (Guo et al., 2020); and (3) damage to intestinal cells through infection must be restored quickly to limit susceptibility to secondary infections and reduce nutrient loss (Teng et al., 2020). Thus, combinations of compounds with supportive functions are expected to increase the ability of the broiler flock to cope with infection, reducing the negative consequences on performance and welfare.

A combination of probiotics (*Enterococcus faecium, Bifidobacterium animalis*, and *Lactobacillus salivarius*) has been shown to alleviate the negative effects of mixed *Eimeria* infection on broiler performance and intestinal integrity (Ritzi et al., 2014). Beta-glucans (Levine et al., 2018) and saponins (Aly et al., 2019) are considered promising candidates to modulate the immune system after *Eimeria* infection because they may act against inflammatory processes in *Eimeria*-infected broilers. Moreover, the extract from *Pleurotus ostreatus*, which is rich in saponins, inhibits *Eimeria* development (Ademola et al., 2019). Tannins were efficient in reducing oocyst shedding in 58% of challenged birds when compared to those that were nontreated, and decreased lesion scores with the same efficiency as compared to the anticoccidial salinomycin (Tonda et al., 2018). Likewise, artemisin (Almeida et al., 2012; Wiedosari and Wardhana, 2017) and curcumin (Kim et al., 2013) counteract the negative effects of an *Eimeria* infection by decreasing oocyst output and lesions, especially in the caecum, and attenuating the severity of the infection. Finally, it is important to recover intestinal function. Butyrate facilitates the recovery of microbiota function (Bortoluzzi et al., 2017) and supports intestinal recovery from lesions (Ali et al., 2014; Zou et al., 2019). Additionally, threonine can support the chickens’ final growth, compensating for the loss of growth efficiency and minimizing losses due to subclinical necrotic enteritis (Star et al., 2012).

Therefore, we hypothesized that combining early applications of probiotics to support gut health before infection (1: intestinal microbiota balance), saponins, beta-glucans, tannins, artemisin, or curcumin when *Eimeria* is abundantly replicating in the hosts (2: decrease in the lesions caused by the infection), and butyrate and threonine in the last phase (3: recovery) could reduce the negative consequences of coccidiosis. In the present study, we evaluated different combinations of these compounds in *Eimeria*-challenged broilers to determine their efficacy in counteracting the negative effects of coccidiosis on performance, gut health, and welfare.

## MATERIALS AND METHODS

### Ethics

The study protocol was approved by the Dutch Central Authority for Scientific Procedures on Animals and the Animal Experiments Committee of Utrecht University (Utrecht, the Netherlands) under registration number AVD246002016766. All procedures were conducted in full compliance with all relevant legislation.

### Broiler Chickens and Housing

A total of 720 one-day-old male Ross 308 (from 38-wk-old broiler breeders) broiler chickens were obtained from a commercial hatchery (Probroed and Sloot B.V., Groenlo, the Netherlands) and housed in 36 floor pens (1.55 × 0.95 m, 20 broilers per pen; 0.07 m^2^/chicken) bedded with white wood shavings (1 kg/m^2^) at the Poultry Research Centre of Coppens Diervoeding B.V. (Helmond, the Netherlands). Chickens without abnormalities were placed in floor pens equipped with 2 feeder bins (150 cm feeding space) and 2 drinking nipples with drip cups. Only at day (**d**) 29, due to wet litter, fresh wood shavings were added (1.3 kg/per pen). Pens were separated by wire mesh panels covered with a cardboard plate, 50 cm in height from the floor, to prevent cross contamination between pens. Each pen was considered a replicate. Ambient temperature in the house gradually decreased from 36°C at arrival to 19°C at d35. On d0, the light was on for 12 h, followed by a dark period of 4 h. Over the next 4 d, a 23**L**(light):1**D**(dark) schedule was applied. Thereafter, the dark period increased by one hour per day, and from d11 onwards, the light schedule was maintained at 15L:4D:1L:4D for the remaining experimental period. Broilers were vaccinated at the hatchery against infectious bronchitis and on d15 and d22 against Newcastle disease and infectious bursal disease (**IBD**), respectively. Drinking water was supplemented with an acidifier (Selko-pH; Selko Feed Additives, Trouw Nutrition, the Netherlands) at a dilution of 1 L acidifier:3 L water throughout the experimental period, except during IBD vaccination on d22. The animals had ad libitum access to feed and water.

### Experimental Design

The experiment used a completely randomized block design with 6 treatment groups and 6 replicate pens per treatment. Treatments were randomly allocated per block (row of 6 pens).

In all treatment groups, birds were inoculated with a mixed *Eimeria* infection on d7, as described below. There were 4 dietary treatments that differed in test components and period of supply, as shown in Table 1. In brief, Treatments (**TRT**) 1 to 4 received a blend of probiotics from d0 to 9 and butyrate and threonine from d28 to 35. In addition, from d9 to 18, TRT 1 received a combination of a blend of probiotics and 1,3/1,6 beta-glucans, followed by a blend of probiotics combined with 1,3/1,6 beta-glucans and tannins from d18 to 28 (referred to as **PRO2-BG2-TAN**). TRT2 received only 1,3/1,6 beta-glucans from d9 to 18, followed by a combination of 1,3/1,6 beta-glucans and artemisin and curcumin from d18 to 28 (**BG2-ART-CUR**). For TRT3, the blend of probiotics was combined with saponins from d9 to 18, followed by a blend of probiotics combined with saponins and tannins from d18 to 28 (**PRO2-SAP2-TAN**). TRT4 received only saponins from d9 to 18, followed by saponins combined with artemisin and curcumin from d18 to 28 (**SAP2-ART-CUR**). The positive control group (**PC**, TRT5) was fed with the standard diet supplemented with the anticoccidial diclazuril. The negative control group (**NC**, TRT6) did not receive a test ingredient or anticoccidial treatment.

**Table 1:** Experimental treatments^1^ in the starter (D0-9), grower 1 (D9-18), grower 2 (D18-28), and finisher (D28-35) phases.

TRT	*Eimeria*^2^	Abbr^3^	D0-9 - starter	D9-18 – grower 1	D18-28- grower 2	D28-35 - finisher
1	YES	PRO2-BG2-TAN	Multispecies probiotic	Multispecies probiotic + 1,3/1,6 beta-glucans	Multispecies probiotic + 1,3/1,6 beta-glucans + tannin	Butyrate + threonine
2	YES	BG2-ART-CUR	Multispecies probiotic	1,3/1,6 beta-glucans	1,3/1,6 beta-glucans + artemisin + curcumin	Butyrate + threonine
3	YES	PRO2-SAP2-TAN	Multispecies probiotic	Multispecies probiotic + Saponins	Multi-species probiotic + Saponins + tannin	Butyrate + threonine
4	YES	SAP2-ART-CUR	Multispecies probiotic	Saponins	Saponins + artemisin + curcumin	Butyrate + threonine
5	YES	PC	Anticoccidial: diclazuril (Clinacox), 1 mg/kg feed			
6	YES	NC	No anticoccidial			

1 Dosages of test compounds in the diet: Multi-species probiotic (*E. faecium + B. animalis + L. salivarius*): 1 g/kg; 1,3/1,6 beta-glucans: 0.5 g/kg; Saponins (Quilaja): 150 mg/kg; Tannin: 100 mg/kg; Artemisin: 35 mg/kg; Curcumin: 35 mg/kg; Butyrate (coated Na-Butyrate): 1 g/kg diet; Threonine level of 0.67%

2 *Eimeria acervulina, E. maxima, and E. tenella* in a dose of 1000, 100 and 500 sporulated oocysts per broiler respectively.

3 Abbr: Abbreviations of the treatments used in grower 1 and 2 diets (as starter and finisher diets are similar for TRT1-4); PC: positive control diet (infected and treated with anticoccidials); NC: negative control, infected and untreated; PRO: probiotics; BG2: 1,3/1,6 beta-glucans; TAN: tannin; ART: artemisin; CUR: curcuma; 2 added; applied in both growers 1 and 2 phase.

### Experimental Diets

The diets were manufactured at the Research Diet Service (Wijk bij Duurstede, the Netherlands). There were 4 feeding phases: a starter period from d0 to 9, grower 1 period from d9 to 18, grower 2 period from d18 to 28, and finisher period from d28 to 35. The basal starter, grower 1, grower 2, and finisher diets were formulated to meet the requirements for all essential nutrients for broilers as recommended by the Nutrients Requirements of Poultry (National Research Council, 1994). The composition and nutrient contents of these diets are provided in Supplementary Table 1. Each of the basal diets was split into six sub-batches, representing the control and supplemented diets. Additive test products were added to the sub-batches according to the dosage levels (Table 1). All diets contained phytase and xylanase. No anticoccidial or other additives were included, except for TRT5 (PC), which contained a chemically synthetized anticoccidial (diclazuril), and TRT1–4, which contained the additive test products, which were additional ingredients. After diet preparation, samples were sent for analysis of the blend of probiotics, saponins, butyrate, and threonine. The presence of these test ingredients in the diets was analyzed with high-performance liquid chromatography (**HPLC**).

### Eimeria Inoculation

On d7, all birds were orally inoculated with 1 mL containing 1,000 sporulated oocysts of *E. acervulina*, 100 of *E. maxima*, and 500 of *E. tenella*. The inoculum was prepared by Gezondheidsdienst voor Dieren (**GD**) Animal Health (Deventer, the Netherlands) on the day of inoculation and contained in-house strains of *E. maxima* and *E. tenella* (both Weybridge strains) maintained by regular passage at GD Animal Health. The *E. acervulina* strain (Brittany strain) was kindly provided by Anses-Laboratoire de Ploufragan-Plouzané (Ploufragan, France).

### Broiler Performance

Broilers were weighed per pen on d0, d9, d18, d28, and d35, and feed consumption per pen was measured on d9, d18, d28, and d35. Mortality was checked and recorded daily. The body weight gain (**BWG**), feed intake (**FI**), and feed conversion ratio (**FCR**) were determined per feeding phase, for the total experimental period until the next feeding phase, and for the total experimental period and corrected for the weight of the birds that died during the experiment as previously shown by Dersjant-Li et al. (2014) with slight modifications. This correction was made by taking the sum of [(number of dead birds on day x) * (weight of dead birds on day x)] for each day in that period.

### Oocyst Shedding

Fecal samples were collected from each pen on d9 to verify the absence of infection before inoculation and on d14, d22, d28, and d35. At least 3 fresh droppings per pen, if available including one cecal dropping, were collected and homogenized. Oocyst shedding per pen was assessed using the sedimentation flotation technique (d9) ( Long et al., 1976) and, on other days, quantified using a modification of the McMaster method (Velkers et al., 2010). *E. maxima* oocysts were visually distinguished from *E. acervulina* and *E. tenella* oocysts. The results are presented as the number of oocysts per gram of feces (**OPG**) for all 3 *Eimeria* species combined (OPG total) and for only *E. maxima* (OPG *E. maxima*).

### Eimeria Lesion Scores and Cecal Weight

On d14, d22, d28, and d35, 2 birds per pen were randomly selected and euthanized by electrocution and exsanguination, and within minutes, their intestinal tract was removed for macroscopic examination. *Eimeria* lesion scores were determined for *E. acervulina, E. maxima*, and *E. tenella* at their respective multiplication sites in the duodenum, jejunum, and caeca. Caeca were collected in the same birds and immediately weighed after collection and prior to lesion scoring. The body weight of each bird was also recorded to calculate the respective relative cecal weight.

### Footpad Lesions and Litter Score

Footpad lesion scores were determined on d32. The left footpads of three randomly selected broiler chickens per pen were scored. The scoring classes were: 0 (no evidence of footpad dermatitis); 1 (mild footpad dermatitis); and 2 (severe footpad dermatitis), according to de Jong et al. (2012). The average score per pen was calculated as:

(% birds with score 0 × 0) + (% birds with score 1 × 0.5) + (% birds with score 2 × 2)

Litter was visually scored per pen on d22, d28, and d35 on a scale from 1 to 5, where 1 represented dry and loose litter and score 5 indicated very wet litter (Haslam et al., 2006).

### Statistical Analyses

Regarding production performance, cecal relative weight, footpad lesions, and litter score, observations were marked as outliers to be excluded from the dataset prior to statistical analyses if the residual (fitted—observed value) was more than 2.5 times the standard error of the parameter. If at least one of the response parameters, feed intake, BWG, or FCR, was an outlier, then all three records were dropped for that observation in that measurement period. The experimental data were analyzed with Analysis of variance (**ANOVA**) (GenStat Version 19.0, 2018) with pen as the experimental unit. Cecal weight and footpad lesions were analyzed using the average per pen (n = 36). The statistical challenge used to analyze the data was:

$$Y=\mu +\text{bloc}k_{i}+\text{Treatmen}t_{j}+e_{\text{ij}}$$

where Y is the response parameter; µ is the general mean; Block_i_ is the effect of row (i = 1…6); Treatment_j_ is the effect of treatment (j = 1…6); and Error_ij_ is the error term.

Treatment means were compared by least significant difference (**LSD**) using Fisher's unprotected LSD test. Values with P ≤ 0.05 were considered statistically significant.

Oocyst count data (OPG), total OPG (all *Eimeria* spp. combined), and *E. maxima* OPG data were analyzed after applying a log₁₀-transformation (log₁₀ [OPG + 1]) to normalize the OPG data. The *lme* function from the *nlme* package in statistical software package R version 3.3.0 (R core team, 2016) was used for linear mixed model analyses. Different models were tested using a stepwise backward approach with single-term deletions. Model selection was based on Akaike's information criterion (**AIC**), with the lowest AIC indicating the best fit. First, a full model was tested containing all potential factors (i.e., ‘Treatment,’ ‘Day,’ and the interaction between Treatment and Day) with the PC diet (anticoccidial treatment) and d14 as reference classes. Pen number was added as a random effect to consider the correlation between repeated measurements, and a constant variance error function (varIdent) was added to model heterogeneity in variances between the days. Model assumptions were evaluated by QQ-plots and a scatterplot of the residuals vs. the predicted values. The statistical model containing Day and Treatment, without the interaction Day x Treatment, was slightly better fitting but the model with Day and Day x Treatment was chosen to obtain estimates to compare the outcomes for the treatments per day of the experiment. Differences in logOPG levels were compared by evaluating the 95% confidence intervals **(95% CI)** around the model estimates. When the interval did not include 0 (i.e., no difference between means of days), it was considered significantly different from the reference category. The same modelling procedure was repeated with the infected and untreated NC diet group as a reference class.

The effect on the likelihood of finding a lesion score for *E. acervulina, E. maxima*, and *E. tenella* lesion scores > 0 was tested with a logistic regression model (procedure *glmer*, package *lme4*, using a binomial distribution and maximum likelihood estimation) with treatment (compared to the PC diet) and scoring day (d22, d28, and d35 vs. d14) as categorical fixed effects. The model including the interaction between Treatment and Day did not converge and was therefore omitted. Pen was added as a random effect to consider the correlation between repeated measurements. The positive control group (**PC**, TRT5) at d14 was used as reference and differences in odds ratios (**OR**) compared to the reference were assessed for significance based on absence of 1 in the 95% CI.

## RESULTS

### Test Ingredient Recovery in the Diets

Diclazuril was recovered in the PC diet (see Supplementary Table 2 for ingredient recovery in the diets). Regarding the test compounds, probiotics were not recovered in the supplemented diets. The analyzed dosages of saponins were mostly higher than expected. Instead of 150 mg saponins/kg diet, the levels were 170 (TRT3, d9–18), 165 (TRT3, d18–28), and 166.7 mg/kg diet (TRT4, d18–28), and levels were lower from d9–18 (106.7 mg/kg diet; TRT4). Coated butyrate (30% butyrate) was added to diets at a dose of 1 g/kg and analysis confirmed this level, as the supplemented diets contained 280 mg/kg butyrate. Threonine, however, was not recovered at the expected level. Diets were formulated to contain 0.64% digestible threonine, but only 0.23% was found after laboratorial analysis (re-analyzed value was 0.36%; Supplementary Table 2).

### Broiler Performance

The average BW of broilers at arrival was 43.0 ± 0.7 g. The results of BW, BWG, FI, and FCR are presented in Table 2. No differences were observed when comparing BWG and FI among treatments, regardless of the feeding period. On d9, a lower FCR was observed in birds fed the PC diet and TRT3 and 4 compared with birds fed the NC diet and TRT1 (*P* < 0.02). On d18, differences were observed only for FCR, with the lowest FCR for the PC and NC (*P* < 0.003). Only broilers fed the TRT1 diet presented an FCR similar to the PC. On d28, all experimental diets had a FCR similar to that of the PC, except for TRT2 (BG2-ART-CUR) and TRT3 (PRO2-SAP2-TAN), which had an increased FCR when compared to the NC (*P* < 0.04). No differences in FCR were observed when comparing the treatments during the finisher period (d28–35). However, when considering the entire experimental period (d0–35), the lowest FCR was observed in PC broilers, followed by TRT4 (SAP2-ART-CUR) and the NC (*P* < 0.001). Mortality rate was similar in all treatments, ranging from 0 to 2.5%.

**Table 2:** Effect of the treatments on broiler chicken average body weight development, feed intake and feed conversion ratio.

	Treatments^1^							
	1	2	3	4	5 (PC)	6 (NC)	LSD	*P*-value
d0–9								
BW D9, g	216	227	221	220	221	218	20.4	0.93
BWG, g	173	184	178	182	179	175	20.0	0.85
FI, g	225	235	222	229	225	229	20.9	0.83
FCR (F:G)	1.306^bc^	1.278abc	1.249^a^	1.259^a^	1.266^ab^	1.313^c^	0.040	0.02
Mortality (%)	0.0	0.0	0.0	0.0	0.0	0.0	*	*
d9–18								
BW D18, g	634	661	644	648	663	659	40.2	0.67
BWG, g	419	434	424	428	442	441	23.4	0.29
FI, g	577	605	590	594	601	595	32.4	0.57
FCR	1.378^bc^	1.395^c^	1.392^c^	1.387^c^	1.360^ab^	1.350^a^	0.023	0.003
Mortality (%)	0.8	0.0	0.0	0.0	0.0	0.0	0.99	0.44
d18–28								
BW D28, g	1374	1385	1322	1383	1414	1432	73.5	0.08
BWG, g	740	725	702	735	735	758	46.4	0.28
FI, g	1089	1127	1091	1085	1098	1088	62.1	0.75
FCR	1.472^ab^	1.559^c^	1.555^bc^	1.477abc	1.496abc	1.436^a^	0.084	0.04
Mortality (%)	1.7	2.5	2.5	1.7	0.8	0.0	3.08	0.53
d28–35								
BW D35, g	2112	2151	2095	2141	2164	2187	116.7	0.63
BWG, g	738	766	773	761	748	756	47.7	0.70
FI, g	1098	1114	1107	1112	1086	1116	62.4	0.92
FCR	1.491	1.454	1.432	1.462	1.452	1.482	0.046	0.15
Mortality (%)	0.0	0.0	0.0	0.8	0.0	0.0	0.99	0.44
d0–35								
BWG, g	2069	2109	2077	2101	2145	2143	109.8	0.61
FI, g	2988	3081	3009	3017	3009	3064	156.3	0.81
FCR	1.445^bc^	1.461^c^	1.449^bc^	1.437^b^	1.404^a^	1.430^b^	0.022	0.001
Mortality (%)	2.5	2.5	2.5	2.5	0.8	0.0	3.44	0.51

a-c Values without a common letter within a column differ significantly (*P* < 0.05)

1 TRT1-4: *Eimeria* infected and subjected to diet treatments 1-4; TRT5:PC: ’positive’ control group 5 (*Eimeria* infected/treated with anticoccidial); TRT6:NC: negative control group 6 (*Eimeria* infected / basal diet no additives).

### Oocyst Counts (OPG)

The descriptive analysis of the mean oocyst counts and standard deviations (**SD**) for the different treatments and day of the experiment are summarized in Table 3. For total OPG (all *Eimeria* spp. combined), as well as for *E. maxima*, the statistical model with Day and Day x Treatment was chosen to obtain estimates to compare outcomes for the treatments per day (Supplementary Table 3 and 4).

**Table 3:** Mean oocyst output (logOPG) and standard deviation (SD) for all *Eimeria* species combined (total) and for *E. maxima* separately for the different treatments and days of the experiment.

		d14				d22			
TRT^1^	Anticoccidial	Log OPG total^2^		Log OPG *E. maxima*^2^		Log OPG total		Log OPG *E. maxima*^2^	
		Mean	SD	Mean	SD	Mean	SD	Mean	SD
1	No	4.78	0.28	4.30	0.43	5.16	0.48	4.47	0.53
2	No	4.93	0.30	4.46	0.27	4.92	0.41	4.23	0.42
3	No	4.71	0.27	4.29	0.29	5.12	0.58	4.51	0.63
4	No	4.79	0.51	4.24	0.46	4.80	0.38	4.15	0.39
5:PC	Yes	3.67	0.57	3.67	0.57	4.30	0.42	4.30	0.42
6:NC	No	4.37	0.39	4.08	0.30	4.71	0.38	4.54	0.24

		d28				d35			
TRT^1^	Anticoccidial	Log OPG total		Log OPG *E. maxima*		Log OPG total		Log OPG *E. maxima*^2^	
		Mean	SD	Mean	SD	Mean	SD	Mean	SD

1	No	3.80	0.44	3.40	0.53	1.10	1.21	0.39	0.95
2	No	4.13	0.43	3.53	0.32	1.36	1.49	0.81	1.25
3	No	3.86	0.58	2.85	1.47	0.93	1.44	0.44	1.08
4	No	3.76	0.27	3.05	0.48	1.12	1.24	1.12	1.24
5:PC	Yes	2.53	0.43	2.53	0.43	0.00	0.00	0.00	0.00
6:NC	No	2.73	1.38	1.47	1.64	2.69	0.46	1.53	1.21

1 TRT1-4: *Eimeria* infected and subjected to diet treatments 1-4; TRT5:PC: ’positive’ control group 5 (*Eimeria* infected/treated with anticoccidial); TRT6:NC: negative control group 6 (*Eimeria* infected/basal diet no additives).

2 The log OPG total includes all oocysts (*E. acervulina, E. maxima* and *E. tenella*). The log OPG *E. maxima* is based on counts of the subset of oocysts in the sample with visibly larger oocysts, recognizable as *E. maxima* oocysts specifically. For total OPG (all *Eimeria* spp. combined), as well as for *E. maxima*, the statistical model with Day and Day x Treatment was chosen to obtain estimates to compare outcomes for the treatments per day (Supplementary Table 3).

The mean total oocyst and *E. maxima* counts generally increased slightly between d14 and d22 and decreased from d28 onwards (Table 3 and Supplementary Table 3 and 4). Differences in mean logOPG among treatments varied across days. On d14, d22, and d28, the total oocyst output was higher in general for most treatments compared to the PC, including the NC, but NC was not significantly different from the PC on d22 and d28 (Table 3 and Supplementary Table 3). On d35, only TRT2 (BG2-ART-CUR) and the NC had a significantly higher total oocyst output compared to the PC (Table 3 and Supplementary Table 3). With the NC as the reference group (Supplementary Table 5), a significantly higher oocyst output was found for TRT2 (BG2-ART-CUR) and a significantly lower output for the PC on d14 (Table 3 and Supplementary Table 5). On d22, no significant differences were found for any of the treatments compared to the NC. On d28, oocyst output was significantly higher for TRT1–4 compared to the NC, but no significant difference was found between the PC and NC. On d35, the oocyst output was lower in all groups, including the PC, compared to the NC (Table 3 and Supplementary Table 5).

When the same analysis as for total logOPG with PC as reference group was done for *E. maxima* oocyst excretion only (Table 3 and Supplementary Table 4), different results were obtained. Differences were mostly found on d14 with TRT1–4 showing a significantly higher *E. maxima* output compared to the PC. On d28, only the NC had a lower *E. maxima* output than the PC, but none of the treatments were significantly different from the PC. On d35, TRT4 (SAP2-ART-CUR) and the NC showed a significantly higher *E. maxima* output (Table 3 and Supplementary Table 4). To assess whether this observed difference between treatment groups in *E. maxima* output also affected the total OPG, the relative contribution of *E. maxima* was calculated (Supplementary Table 6). For the PC, 100% of the total OPG was due to *E. maxima* throughout the experiment. For TRT1–4 and the NC, the output varied between 86 and 96% on d14 and d22, while on d28, it was between 54 (NC) and 89%. The largest treatment differences were found on d35, with 100% of the output due to *E. maxima* for TRT4 (SAP2-ART-CUR), whereas for TRT1–3 and the NC this varied from 35 to 60%.

### Eimeria Lesion Scores

The descriptive analyses describing the mean lesion scores and SD for *E. acervulina, E. maxima* and *E. tenella* are shown in Table 4. For the logistic models of the *E. acervulina* and *E. tenella* lesion scores, Treatment was a significant factor (Table 5 and 7), but not for *E. maxima* (Table 6), but was retained in all models to obtain OR estimates for the different treatments compared to the PC (Table 5, Table 6, Table 7). Broilers receiving TRT4 (SAP2-ART-CUR) generally showed lower mean lesion scores (Table 4 for all *Eimeria* spp. compared with the other treatments and either showed a significantly reduced risk for lesions or no higher risk for lesions compared to the PC (Table 5, Table 6, Table 7). The risk of having an *E. acervulina* lesion score above 0 was 0.18 times as high (i.e., 5 times lower) for TRT4 (SAP2-ART-CUR) compared to the PC (Table 5). In general, the risk of *E. acervulina* lesions was significantly higher on d28 and d35 compared to d14. For *E. maxima* (Table 6), no treatment effects were shown, but a progressive increase in the risk of lesions on d22, d28, and d35 was observed. For *E. tenella*, only a significant increase in the presence of lesions was observed for d22 compared to d14 (Table 7).

**Table 4:** Mean *Eimeria* lesion scores and standard deviation (SD) for *E. acervulina* (EA), *E. maxima* (EM) and *E. tenella* (ET) for the different treatments and days of the experiment.

		*Eimeria* lesion scores for d14					
TRT^1^	Anticoccidial	Lesion scores EA		Lesion scores EM		Lesion scores ET	
		Mean	SD	Mean	SD	Mean	SD
1	No	0.42	0.52	0.33	0.49	0.29	0.45
2	No	0.33	0.49	0.58	0.69	0.67	0.89
3	No	0.17	0.39	0.17	0.39	0.36	0.77
4	No	0.08	0.29	0.25	0.45	0.49	0.50
5:PC	Yes	0.08	0.29	0.25	0.62	0.33	0.75
6:NC	No	0.25	0.45	0.00	0.00	0.33	0.65

		*Eimeria* lesion scores for d22					
TRT^1^	Anticoccidial	Lesion scores EA		Lesion scores EM		Lesion scores ET	
		Mean	SD	Mean	SD	Mean	SD

1	No	0.50	0.52	0.25	0.45	1.71	1.01
2	No	0.33	0.49	0.75	0.86	2.08	0.9
3	No	0.25	0.45	0.67	0.49	2.17	0.94
4	No	0.08	0.29	0.58	0.67	1.58	1.24
5:PC	Yes	0.42	0.52	0.58	0.9	0.25	0.62
6:NC	No	0.25	0.45	0.58	0.67	0.38	0.64

		*Eimeria* lesion scores for d28					
TRT^1^	Anticoccidial	Lesion scores EA		Lesion scores EM		Lesion scores ET	
		Mean	SD	Mean	SD	Mean	SD

1	No	0.92	0.29	1.25	0.62	0.33	0.49
2	No	0.79	0.40	1.17	0.72	0.67	0.96
3	No	0.75	0.62	1.33	0.65	0.75	0.97
4	No	0.50	0.52	0.54	0.75	0.13	0.31
5:PC	Yes	1.08	0.29	1.5	0.67	0.42	0.47
6:NC	No	0.79	0.40	0.83	0.84	0.33	0.49

		*Eimeria* lesion scores for d35					
TRT^1^	Anticoccidial	Lesion scores EA		Lesion scores EM		Lesion scores ET	
		Mean	SD	Mean	SD	Mean	SD

1	No	0.38	0.23	0.67	0.49	0.42	0.9
2	No	0.42	0.2	0.83	0.39	0.33	0.44
3	No	0.58	0.2	0.83	0.58	0.83	0.94
4	No	0.25	0.34	0.67	0.49	0.17	0.39
5:PC	Yes	0.42	0.36	1.08	0.29	0.08	0.29
6:NC	No	0.50	0.21	1.33	0.49	0.29	0.45

1 TRT1-4: *Eimeria* infected and subjected to diet treatments 1-4; TRT5:PC: ’positive’ control group 5 (*Eimeria* infected/treated with anticoccidial); TRT6:NC: negative control group 6 (*Eimeria* infected/basal diet no additives).

**Table 5:** Model estimates for the odds ratio (OR) and lower and upper values for the 95% interval around the OR for the lesion scores model for *E. acervulina* with the positive control (PC, TRT5) as reference*.*

	Estimate^1^	95% confidence interval		
	expB (OR)	2.5%	97.5%	Sign
(Intercept) (d14/TRT 5: PC)	0.26	0.09	0.72	
d14: TRT1	1.93	0.55	6.73	
d14: TRT2	1.29	0.37	4.48	
d14: TRT3	0.88	0.25	3.03	
d14: TRT4	0.18	0.05	0.64	*
d14: TRT6	1.14	0.33	3.94	
d22	1.63	0.74	3.63	
d28	21.77	8.51	55.70	*
d35	17.93	7.20	44.62	*

1 Values with * in last column were significantly different compared to the reference category (positive control, TRT 5), based on absence of 1 in the 95% confidence interval.

**Table 6:** Model estimates for the odds ratio (OR) and lower and upper values for the 95% interval around the OR for the lesion scores model for *E. maxima*^2^ with the positive control (PC, TRT5) as reference*.*

	Estimate^1^	95% conference interval		
	expB (OR)	2.5%	97.5%	Sign
(Intercept) (d14/TRT 5: PC)	0.38	0.17	0.85	
d14: TRT1	0.64	0.25	1.62	
d14: TRT2	1.44	0.55	3.77	
d14: TRT3	1.00	0.39	2.58	
d14: TRT4	0.46	0.18	1.17	
d14: TRT6	0.57	0.22	1.45	
d22	3.18	1.54	6.58	*
d28	12.29	5.55	27.26	*
d35	16.04	7.00	36.76	*

1 Values with * in last column were significantly different compared to the reference category (positive control, TRT5), based on absence of 1 in the 95% confidence interval.

2 Note that Treatment was not a significant factor in the logistic model for *E. maxima* but was retained in the model to obtain the estimates and compare treatments.

**Table 7:** Model estimates for the odds ratio (OR) and lower and upper values for the 95% interval around the OR for the lesion scores model for *E. tenella* with the positive control (PC, TRT5) as reference*.*

	Estimate^1^	95% confidence interval		
	expB (OR)	2.5%	97.5%	Sign
(Intercept) (d14/TRT 5: PC)	0.21	0.09	0.52	
d14: TRT1	2.90	1.05	8.03	*
d14: TRT2	4.65	1.67	12.95	*
d14: TRT3	4.24	1.52	11.79	*
d14: TRT4	2.17	0.78	6.03	
d14: TRT6	1.43	0.51	4.04	
d22	4.30	2.06	8.98	*
d28	1.14	0.56	2.33	
d35	0.76	0.36	1.58	

1 Values with * in last column were significantly different compared to the reference category (positive control, TRT5), based on absence of 1 in the 95% confidence interval.

### Relative Cecal Weight

Birds of the NC and PC groups had the highest relative cecal weight on d14 and d22, as represented in Table 8 (*P* < 0.01). On d28, birds of TRT4 (SAP2-ART-CUR) had a similar relative cecal weight to that observed in birds of the NC and PC groups. On d35, no differences were observed among the treatments in relative cecal weight.

**Table 8:** Effect of different treatments on relative (%) cecal weight.

TRT^1^	Anticoccidial	d14		d22		d28		d35	
1	No	0.78^a^		0.56^a^		0.57abc		0.81	
2	No	0.75^a^		0.55^a^		0.48^a^		0.78	
3	No	0.70^a^		0.52^a^		0.57^ab^		0.69	
4	No	0.79^a^		0.63^a^		0.72bcd		0.59	
5	Yes	1.19^b^		0.98^b^		0.79^d^		0.72	
6	No	1.02^b^		0.88^b^		0.78^cd^		0.87	
		*P*-value	LSD	*P*-value	LSD	*P*-value	LSD	*P*-value	LSD
		*<0.001*	0.229	*<0.001*	0.161	*0.023*	0.211	0.085	0.192

a-d Values without common letters within a column differ significantly (*P* < 0.05).

1 TRT1-4: *Eimeria* infected and subjected to diet treatments 1-4; TRT5:PC: ’positive’ control group 5 (*Eimeria* infected/treated with anticoccidial); TRT6:NC: negative control group 6 (*Eimeria* infected/basal diet no additives).

### Footpad Lesions and Litter Score

No differences among treatments were observed when comparing footpad lesions or litter quality (Supplementary Table 7).

## DISCUSSION

We hypothesized that *Eimeria*-challenged broilers would benefit from the use of a combination of nutritional ingredients provided in the phase of the infection where it could most optimally exert its effect. In this experiment we aimed to optimize gut health prior to infection, to target immune function, and invasion and multiplication of *Eimeria* spp. in the early stages of infection, and to apply intestinal function restoring ingredients during and after peak infection. Together, these nutritional ingredients could reduce the negative effects of coccidiosis infection on performance, gut health, and welfare parameters in broiler chickens. Specific combinations of dietary additives, as tested in this study, did however not result in better performance or better litter quality and fewer footpad lesions compared to the anticoccidial-treated PC group. Additionally, the PC was most effective in reducing oocyst output and lesion scores. Yet, some differences in FCR, oocyst output, and lesion scores between dietary treatments were found.

The effectiveness of the selected probiotics could not be evaluated because this additive was not sufficiently recovered in the diet, which may explain the absence of a difference between challenged birds supplemented with probiotics and the nontreated NC group in the starter period. This discrepancy between calculated and recovered probiotics indicates that it might be necessary to analyze the actual content of probiotics in terms of colony forming units (**CFU**) before mixing the diets. Additionally, a blend of free and buffered organic acids (Selko-pH, Trouw Nutrition) was present in the water of all birds. It is unlikely, however, that this organic acid blend in the water could have affected the probiotic strains at the gut level. The probiotic strains were selected based on their stability against acids and bile salts, as they also need to withstand the low pH in the gizzard. Moreover, their stability in combination with organic acids has been previously confirmed (Pender et al., 2020). Organic acids do not have direct effects on *Eimeria* survival or multiplication on intestinal level, but may support microbial balance, reduce the impact of *Eimeria* infections, and increase BWG (Peek and Landman, 2011). However, as the blend of organic acids was provided in all groups, effects on the study outcomes are not likely.

In some studies, more challenging situations with high *Eimeria* infection levels and combinations with *Clostridium perfringens* resulted in necrotic enteritis (Adhikari et al., 2020) and are used to evaluate the effectiveness of alternatives for in-feed antimicrobials (Granstadt et al. (2020)). In this study, we used a relatively mild challenge with the 3 most relevant *Eimeria* spp. in broilers, that is, *E. acervulina, E. maxima*, and *E. tenella*, and applied it at an early age. Although clinical coccidiosis, sometimes followed by necrotic enteritis, does occur in commercial farms, on many farms, coccidiosis can remain subclinical. Moreover, flock infections start with relatively low doses at an early age, before chickens are exposed to high oocyst levels later in the production period (Klinkenberg and Heesterbeek, 2007; Chapman et al., 2016; Snyder et al., 2021), making our infection model compliant to field conditions.

However, differences in production performance between the NC and PC remained limited, with only a significant difference during the starter period (d0–9) and overall feeding period (d0–35). Most dietary strategies, as tested in the present experiment, even under this relatively mild challenge, were also not able to achieve significantly better results than the NC regarding performance, OPG counts, and intestinal lesions. The experimental farm where this study was performed mimicked field conditions. However, the bacterial and viral intestinal pathogens load and climate and feed quality may have been slightly better compared to large commercial farms. This may in part explain the relative small difference in performance for the PC as compared to the NC, as the growth promoting and antibacterial effects of the PC treatment may be more pronounced in a more challenging environment.

Cecal weight is related to fermentation activity (Yang et al., 2020), and it is negatively correlated to the amount of water excreted, meaning that a well-developed caecum contributes to a lower amount of water loss through excreta (Svihus et al., 2013) and could result in better litter quality. Although we observed multiple differences in cecal weight between treatments, we did not detect a correlation between cecal weight and litter quality in the present study. In all treatments, the litter was rather wet, which could have masked any treatment differences in visual quality.

In all experimental groups, oocyst excretion peaked on d22 and declined after d28, which is consistent with the *Eimeria* infection dynamics in broiler flocks as a result of increasing immunity (Jenkins et al., 2017; Snyder et al., 2021). On d14, d22, and d28, the total oocyst output was significantly lower in the PC compared to all treatments, except for the NC, suggesting that the dietary treatments as tested here had limited effects on reducing peak oocyst excretion. However, at the end of the trial on d35, oocyst output was lower in all experimental treatments compared to the NC. This may indicate that the development of immunity was more efficient in TRT1–4 compared to the NC, which can either be a possible delayed effect of TRT1–4 or a result of the higher oocyst excretion earlier in the production period, which may stimulate immune development (Klinkenberg and Heesterbeek, 2007).

*Eimeria* spp., other than *E. maxima,* were more effectively controlled by both the anticoccidial and dietary treatments. The high percentage of *E. maxima* in the total oocyst output after d14 suggests that the inoculation for *E. maxima* may have resulted in a relatively more severe infection compared to *E. acervulina* or *E. tenella*, despite the low dosages (100 sporulated oocysts for *E. maxima* vs. 1000 for *E. acervulina* and 500 for *E. tenella*). As *E. maxima* could not be controlled sufficiently by the different treatments, including the PC, and with *E. maxima* generally having the most impact on body weight and FCR (Kipper et al., 2013), this may explain the lack of measurable effects on performance parameters. The limited effectiveness of different anticoccidials against several *Eimeria* spp. is well known (Noack et al., 2019). However, the findings were surprising because diclazuril is known for its ability to impair the wall synthesis of oocysts from *E. maxima* (Verheyen et al., 1989) and was also recovered in the targeted dose in the diet. For *Eimeria* spp., other than *E. maxima*, differences in the efficacy of the treatments were observed, with the PC being very effective against the other species present in the inoculum. The combination of saponins, curcumin, and artemisin (TRT4, SAP2-ART-CUR) seemed to be able to completely reduce the output of the other species by d35 but not for *E. maxima*, whereas the NC group had an almost equal quantity of other species and *E. maxima* after d28. The beneficial effects of TRT4 (SAP2-ART-CUR) on reducing oocyst output on species other than *E. maxima* would have remained undetected when the relative contribution of different *Eimeria* spp. to total oocyst output would not have been evaluated. Thus, this approach is recommended for efficacy studies to fully evaluate the effect of treatments on oocyst excretion dynamics.

Similarly, TRT4 (SAP2-ART-CUR) also had lower lesion scores for all species, and either showed a reduced (*E. acervulina* and *E. maxima*, although for *E. maxima* only a tendency was found) or equal risk for lesions (*E. tenella*) compared to the NC. This indicates that for all treatments other than the PC, TRT4 (SAP2-ART-CUR) best reduced oocyst shedding and lesions, at least for *E. acervulina* and *E. tenella*. Artemisin can decrease *E. tenella* infections (Dragan et al., 2014), but a 100-fold higher dose is necessary to achieve success similar to *E. acervulina* and *E. maxima* (Pop et al., 2015). In a study by Almeida et al. (2014), the combination of curcumin and artemisin in the diets of challenged broiler chickens was effective against *E. acervulina* but not against *E. maxima*. In our study, the additional inclusion of saponins did not improve the effectiveness against *E. maxima*, but the combination of these three compounds decreased both oocyst output and intestinal lesions, indicating that the combination of artemisin, curcumin, and saponin had anticoccidial activity. Saponins can bind sterol molecules present on the cell membrane of parasites, resulting in oocyst lysis, whereas curcumin not only acts against the parasites but also enhances host humoral immunity (Muthamilselvan et al., 2016). Artemisin directly inhibits sporulation and cell wall formation by inducing oxidative stress in *Eimeria* spp. (Muthamilselvan et al., 2016).

Although not different from the NC, TRT1 (PRO2-BG2-TAN) resulted in a better FCR than TRT2 (BG2-ART-CUR) and TRT3 (PRO2-SAP2-TAN). Beta-glucans serve as a substrate for microbiota, favoring the adhesion of beneficial bacteria in the intestinal mucosa (Wang et al., 2016). Tannin is a polyphenol with antimicrobial, antiparasitic, antioxidant, anti-inflammatory, and antivirus effects (Sugirharto, 2016). Its molecular structure favors the formation of complexes with small and large molecules, including polysaccharides, such as beta-glucans (Serrano et al., 2009). When the tannin and beta-glucan complex was studied in vitro, it showed pH and temperature dependency, where an increase in pH from 2 to 9 and a temperature increase from 20 to 90°C had a negative effect on the presence of these complexes (Li et al., 2019). Therefore, intestinal pH and feed processing (pelleting) may have negatively affected this type of interaction in the present study. Finally, one could suggest that butyrate combined with threonine supported broiler recovery, as observed by a decrease in the performance differences among TRT1 (PRO2-BG2-TAN) and TRT4 (SAP2-ART-CUR)) compared to TRT2 (BG2-ART-CUR) and TRT3 (PRO2-SAP2-TAN). The analyzed threonine levels were even lower than the expected levels when threonine was not added to the diet, because threonine is also delivered by feedstuffs. Besides, all diets were supplemented with threonine to reach 0.64% and 0.67% in the finisher phase without or with extra threonine addition. Although threonine recovery analysis did not show the intended dietary levels, it can be argued that the levels of this amino acid were not at a deficient level. This is supported by a growth similar to that of the broiler chickens from PC group.

The combinations of dietary additives showed limited effects in our study design, even though the compounds and period of administration were selected based on previously published studies showing positive effects on broiler performance, gut health, and anticoccidial effectivity. Perhaps less favorable outcomes with these compounds have not been published due to so-called publication bias (Blajman et al., 2014) and differences in study design or unknown factors.

### Conclusions

The best FCR was obtained in the PC group, and none of the combinations of dietary additives resulted in a better BWG and FCR than the NC group. However, the combination of saponins, artemisin, and curcumin during the grower 2 period in TRT4 reduced oocyst output and lesion scores.

## Appendix Supplementary materials

Image, application 1

Image, application 2

Image, application 3

Image, application 4

Image, application 5

Image, application 6

Image, application 7

## Footnotes

Supplementary material associated with this article can be found in the online version at doi:10.1016/j.psj.2022.101853.
