# Supplementary material for: Nutritional interventions to support broiler chickens during Eimeria infection
Source: Poult Sci. 2022 Mar 11;101(6):101853. doi: 10.1016/j.psj.2022.101853 (PMC9018146; doi:10.1016/j.psj.2022.101853)
Supplement: Supplementary file 1 [file mmc1.docx]

**Supplementary Table 1.** Composition and calculated nutrient content of the diets

| **Ingredients (%)** |  | **Starter**  **d0-9** | **Grower 1**  **d9-18** | **Grower 2**  **d18-28** | **Finisher**  **d28-35** |
| --- | --- | --- | --- | --- | --- |
| Wheat |  | 35.22 | 42.17 | 48.16 | 54.13 |
| Maize |  | 30.00 | 25.00 | 20.00 | 15.00 |
| Soybean meal |  | 26.94 | 24.35 | 22.46 | 20.53 |
| Sunflowerseed meal |  | 1.00 | 1.50 | 2.00 | 2.50 |
| Soybean oil |  | 2.84 | 3.64 | 4.35 | 5.06 |
| Limestone |  | 1.55 | 1.26 | 1.15 | 0.97 |
| Monocalcium phosphate |  | 0.47 | 0.11 | 0.005 | - |
| NaCl |  | 0.08 | 0.08 | 0.08 | 0.09 |
| Sodiumbicarbonate |  | 0.47 | 0.39 | 0.39 | 0.38 |
| Premix (vit/min/phytase/NSP) |  | 0.50 | 0.50 | 0.50 | 0.50 |
| L-Lysine |  | 0.38 | 0.38 | 0.37 | 0.35 |
| DL-Methionine |  | 0.32 | 0.30 | 0.27 | 0.23 |
| L-Threonine |  | 0.13 | 0.13 | 0.12 | 0.12 |
| L-Arginine |  | 0.08 | 0.08 | 0.07 | 0.06 |
| L-Valine |  | 0.08 | 0.08 | 0.07 | 0.06 |
| **Nutrient composition (g/kg)** |  |  |  |  |  |
| Metabolic Energy (kcal/kg) |  | 2,900 | 2,975 | 3,025 | 3,075 |
| Moisture |  | 120 | 121 | 121 | 122 |
| Crude ash |  | 61 | 54 | 52 | 49 |
| Crude protein |  | 201 | 195 | 190 | 184 |
| Crude fat |  | 46 | 55 | 62 | 70 |
| Crude fibre |  | 20 | 20 | 21 | 21 |
| Starch |  | 415 | 421 | 422 | 424 |
| Ca |  | 9.5 | 7.8 | 7.2 | 6.5 |
| P |  | 6.4 | 5.5 | 5.3 | 5.0 |
| Ret.P (intens) |  | 3.8 | 3.1 | 2.9 | 2.6 |
| Phytase (FTU/kg) |  | 500 | 500 | 500 | 500 |
| K |  | 8.6 | 8.3 | 8.0 | 7.8 |
| Na |  | 1.5 | 1.5 | 1.5 | 1.5 |
| Cl |  | 1.7 | 1.7 | 1.7 | 1.7 |
| sidLYS |  | 11.5 | 11.0 | 10.5 | 10.0 |
| sidMET |  | 5.7 | 5.5 | 5.0 | 4.7 |
| sidM+C |  | 8.3 | 8.0 | 7.6 | 7.2 |
| sidTHR |  | 7.1 | 6.9 | 6.6 | 6.4 |
| sidTRP |  | 2.1 | 2.0 | 2.0 | 2.0 |
| sidILE |  | 7.1 | 6.8 | 6.6 | 6.4 |
| sidARG |  | 12.1 | 11.6 | 11.1 | 10.7 |
| sidVAL |  | 8.5 | 8.1 | 7.9 | 7.6 |

sid: standardized ileal digestibility

^1^ Vitamin and mineral premix supplied per kg diet (based on a 0.5% dose): Vitamin A 10,000 IU; vitamin D3 3,000 IU; vitamin E 150 mg; vitamin K3 3.0 mg; vitamin B1 3.0 mg; vitamin B2 7.5 mg; vitamin B6 4.0 mg; vitamin B12 30 mcg; Niacin 45 mg; D-pantothenic acid 10 mg; Choline chloride 500 mg; Folic acid 1.0 mg; Biotin 0.05 mg; Fe 80 mg (as FeSO_4_.H_2_O);

Cu 15 mg (as CuSO_4_.5H_2_O); Mn 70 mg (as MnO); Zn 90 mg (as ZnSO_4_.H_2_O); I 2.0 mg (as Ca(IO_3_)_2_); Se 0.25 mg (as Na_2_SeO_3_). Phytase (Axtra^(R)^ PHY 10000), Ronozyme WX (50 mg/kg).
