# Supplementary material for: Nutritional interventions to support broiler chickens during Eimeria infection
Source: Poult Sci. 2022 Mar 11;101(6):101853. doi: 10.1016/j.psj.2022.101853 (PMC9018146; doi:10.1016/j.psj.2022.101853)
Supplement: Supplementary file 2 [file mmc2.docx]

**Supplementary Table 2.** Test ingredient recovery in the diets

|  | **Starter** | **Grower 1** | **Grower 2** | **Finisher** |
| --- | --- | --- | --- | --- |
| Diclazuril (g/kg; TRT5) |  |  |  |  |
| calculated | 1.0 | 1.0 | 1.0 | 1.0 |
| analysed | 1.7 | 1.2 | 1.0 | 0.8 |
| Multi-species probiotic (mg/kg) |  |  |  |  |
| calculated | 1.0 | 1.0 | 1.0 | NA |
| analysed | NR | NR | NR | NA |
| 1,3/1,6 beta-glucans (g/kg) |  |  |  |  |
| calculated | NA | 0.5 | 0.5 | NA |
| analysed | NA | Not measured | Not measured | NA |
| Tannin (mg/kg) |  |  |  |  |
| calculated | NA | 100 | 100 | NA |
| analysed | NA | Not measured | Not measured | NA |
| Artemisin (mg/kg) |  |  |  |  |
| calculated | NA | 35 | 35 | NA |
| analysed | NA | Not measured | Not measured | NA |
| Curcumin (mg/kg) |  |  |  |  |
| calculated | NA | 35 | 35 | NA |
| analysed | NA | Not measured | Not measured | NA |
| Saponins (mg/kg) |  |  |  |  |
| calculated | NA | 150 | 150 | NA |
| analysed | NA | 106.7-170^1^ | 165-166.7^2^ | NA |
| Butyrate^3^ (mg/kg) |  |  |  |  |
| calculated | NA | NA | NA | 300 |
| analysed | NA | NA | NA | 280 |
| Threonine (total, %) |  |  |  |  |
| calculated | NA | NA | NA | 0.79 |
| analysed | NA | NA | NA | 0.23/0.36 |

NA: Not applicable; NR: Not recovered; ^1^ 170 mg/kg in TRT3 and 106.7 mg/kg in TRT4; ^2^ 165 mg/kg in TRT3 and 166.7 mg/kg in TRT4; ^3^ 30% Butyrate to obtain 300 mg/kg diet, diets were supplemented with 1 g/kg coated butyrate.
