# Supplementary material for: Nutritional interventions to support broiler chickens during Eimeria infection
Source: Poult Sci. 2022 Mar 11;101(6):101853. doi: 10.1016/j.psj.2022.101853 (PMC9018146; doi:10.1016/j.psj.2022.101853)
Supplement: Supplementary file 3 [file mmc3.docx]

**Supplementary Table 3.** Mean log OPG model estimates and lower and upper 95% interval around estimate for all *Eimeria* species combined (total) with the positive control group (PC, TRT5) as reference

|  | **Estimate^1^** | **95% confidence interval** | |  |
| --- | --- | --- | --- | --- |
|  | **Mean logOPG total** | **Lower limit** | **Upper limit** | **Sign** |
| (Intercept) (d14/TRT 5: PC) | 3.67 | 3.37 | 3.97 |  |
| TRT 5: d22 | 0.63 | 0.18 | 1.08 | * |
| TRT 5: d28 | -1.14 | -1.74 | -0.55 | * |
| TRT 5: d35 | -3.67 | -4.55 | -2.79 | * |
| TRT 1: d14 | 1.11 | 0.69 | 1.54 | * |
| TRT 2: d14 | 1.26 | 0.83 | 1.68 | * |
| TRT 3: d14 | 1.04 | 0.62 | 1.47 | * |
| TRT 4: d14 | 1.12 | 0.70 | 1.54 | * |
| TRT 6: d14 | 0.70 | 0.28 | 1.12 | * |
| TRT 1: d22 | 0.86 | 0.39 | 1.33 | * |
| TRT 2: d22 | 0.62 | 0.15 | 1.09 | * |
| TRT 3: d22 | 0.83 | 0.36 | 1.29 | * |
| TRT 4: d22 | 0.50 | 0.03 | 0.97 | * |
| TRT 6: d22 | 0.41 | -0.06 | 0.88 |  |
| TRT 1: d28 | 1.27 | 0.55 | 2.00 | * |
| TRT 2: d28 | 1.61 | 0.88 | 2.33 | * |
| TRT 3: d28 | 1.33 | 0.61 | 2.06 | * |
| TRT 4: d28 | 1.24 | 0.51 | 1.96 | * |
| TRT 6: d28 | 0.20 | -0.52 | 0.93 |  |
| TRT 1: d35 | 1.10 | -0.08 | 2.27 |  |
| TRT 2: d35 | 1.36 | 0.18 | 2.53 | * |
| TRT 3: d35 | 0.93 | -0.24 | 2.10 |  |
| TRT 4: d35 | 1.12 | -0.06 | 2.29 |  |
| TRT 6: d35 | 2.69 | 1.52 | 3.87 | * |

^1^ Values with * in last column were significantly different compared to the reference category (positive control, PC, TRT5), based on absence of 0 in the 95% confidence interval.
