# Supplementary material for: Nutritional interventions to support broiler chickens during Eimeria infection
Source: Poult Sci. 2022 Mar 11;101(6):101853. doi: 10.1016/j.psj.2022.101853 (PMC9018146; doi:10.1016/j.psj.2022.101853)
Supplement: Supplementary file 4 [file mmc4.docx]

**Supplementary Table 4.** Mean logOPG model estimates and lower and upper 95% interval around estimate for *E. maxima* with the positive control (PC, TRT5) as reference

|  | **Estimate^1^** | **95% confidence interval** | |  |
| --- | --- | --- | --- | --- |
|  | **Mean logOPG** | **Lower limit** | **Upper limit** | **Sign** |
| (Intercept) (D14/TRT 5: PC) | 3,67 | 3,37 | 3,97 |  |
| TRT5: d22 | 0,63 | 0,18 | 1,08 | * |
| TRT5: d28 | -1,14 | -1,92 | -0,36 | * |
| TRT5: d35 | -3,67 | -4,50 | -2,83 | * |
| TRT1: d14 | 0,64 | 0,21 | 1,06 | * |
| TRT2: d14 | 0,79 | 0,37 | 1,21 | * |
| TRT3: d14 | 0,62 | 0,20 | 1,04 | * |
| TRT4: d14 | 0,57 | 0,15 | 0,99 | * |
| TRT6: d14 | 0,41 | -0,01 | 0,83 | ns |
| TRT1: d22 | 0,17 | -0,30 | 0,65 | ns |
| TRT2: d22 | -0,07 | -0,55 | 0,41 | ns |
| TRT3: d22 | 0,21 | -0,27 | 0,69 | ns |
| TRT4: d22 | -0,15 | -0,63 | 0,32 | ns |
| TRT6: d22 | 0,24 | -0,24 | 0,72 | ns |
| TRT1: d28 | 0,87 | -0,14 | 1,89 | ns |
| TRT2: d28 | 1,01 | -0,01 | 2,03 | ns |
| TRT3: d28 | 0,32 | -0,69 | 1,34 | ns |
| TRT4: d28 | 0,53 | -0,49 | 1,54 | ns |
| TRT6: d28 | -1,06 | -2,07 | -0,04 | * |
| TRT1: d35 | 0,39 | -0,72 | 1,49 | ns |
| TRT2: d35 | 0,81 | -0,30 | 1,91 | ns |
| TRT3: d35 | 0,44 | -0,66 | 1,54 | ns |
| TRT4: d35 | 1,12 | 0,02 | 2,22 | * |
| TRT6: d35 | 1,53 | 0,43 | 2,63 | * |

^1^ Values with * in last column were significantly different compared to the reference category (positive control, TRT 5), based on absence of 0 in the 95% confidence interval.
