# Supplementary material for: Nutritional interventions to support broiler chickens during Eimeria infection
Source: Poult Sci. 2022 Mar 11;101(6):101853. doi: 10.1016/j.psj.2022.101853 (PMC9018146; doi:10.1016/j.psj.2022.101853)
Supplement: Supplementary file 5 [file mmc5.docx]

**Supplementary Table 5.** Mean log OPG model estimates and lower and upper 95% interval around estimate for all *Eimeria* species combined (total) with the negative control group (NC, TRT6) as reference

|  | **Estimate^1^** | **95% confidence interval** | |  |
| --- | --- | --- | --- | --- |
|  | **Mean logOPG** | **Lower limit** | **Upper limit** | **Sign** |
| (Intercept) (d14/TRT6: NC) | 4.37 | 4.07 | 4.67 |  |
| TRT6: d22 | 0.34 | -0.11 | 0.79 |  |
| TRT6: d28 | -1.64 | -2.23 | -1.05 | * |
| TRT6: d35 | -1.68 | -2.56 | -0.79 | * |
| TRT1: d14 | 0.41 | -0.01 | 0.84 |  |
| TRT2: d14 | 0.56 | 0.13 | 0.98 | * |
| TRT3: d14 | 0.34 | -0.08 | 0.77 |  |
| TRT4: d14 | 0.42 | 0.00 | 0.84 |  |
| TRT5: d14 | -0.70 | -1.12 | -0.28 | * |
| TRT1: d22 | 0.45 | -0.02 | 0.92 |  |
| TRT2: d22 | 0.21 | -0.26 | 0.68 |  |
| TRT3: d22 | 0.42 | -0.05 | 0.88 |  |
| TRT4: d22 | 0.09 | -0.38 | 0.56 |  |
| TRT5: d22 | -0.41 | -0.88 | 0.06 |  |
| TRT1: d28 | 1.07 | 0.35 | 1.79 | * |
| TRT2: d28 | 1.40 | 0.68 | 2.13 | * |
| TRT3: d28 | 1.13 | 0.40 | 1.85 | * |
| TRT4: d28 | 1.04 | 0.31 | 1.76 | * |
| TRT5: d28 | -0.20 | -0.93 | 0.52 |  |
| TRT1: d35 | -1.60 | -2.77 | -0.42 | * |
| TRT2: d35 | -1.34 | -2.51 | -0.16 | * |
| TRT3: d35 | -1.76 | -2.94 | -0.59 | * |
| TRT4: d35 | -1.58 | -2.75 | -0.40 | * |
| TRT5: d35 | -2.69 | -3.87 | -1.52 | * |

^1^ Values with * in last column were significantly different compared to the reference category (negative control, TRT 6), based on absence of 0 in the 95% confidence interval.
