# Supplementary material for: Nutritional interventions to support broiler chickens during Eimeria infection
Source: Poult Sci. 2022 Mar 11;101(6):101853. doi: 10.1016/j.psj.2022.101853 (PMC9018146; doi:10.1016/j.psj.2022.101853)
Supplement: Supplementary file 6 [file mmc6.docx]

**Supplementary Table 6.** Percentage of logOPG for *E. maxima* of the log OPG for all *Eimeria* species combined (total) for the different treatments and days of the experiment

| **TRT** | **Anticoccidial** | **Percentage of logOPG max of logOPG total** | | | |
| --- | --- | --- | --- | --- | --- |
|  |  | **d14** | **d22** | **d28** | **d35** |
| 1 | No | 90 % | 87 % | 89 % | 35 % |
| 2 | No | 91 % | 86 % | 86 % | 60 % |
| 3 | No | 91 % | 88 % | 74 % | 47 % |
| 4 | No | 89 % | 86 % | 81 % | 100 % |
| 5:PC | Yes | 100 % | 100 % | 100 % | no output |
| 6:NC | No | 93 % | 96 % | 54 % | 57 % |
