# Supplementary material for: Nutritional interventions to support broiler chickens during Eimeria infection
Source: Poult Sci. 2022 Mar 11;101(6):101853. doi: 10.1016/j.psj.2022.101853 (PMC9018146; doi:10.1016/j.psj.2022.101853)
Supplement: Supplementary file 7 [file mmc7.docx]

**Supplementary Table 7**. Effect of different treatments on footpad dermatitis and litter score

|  |  | **Footpad dermatitis** | | |  | **Litter score** | | | | | |
| --- | --- | --- | --- | --- | --- | --- | --- | --- | --- | --- | --- |
| **TRT** | **Anticoccidial** | **d32** | | |  | **d22** | | **d28** | | **d35** | |
| 1 | No |  | 0.75 |  |  | 3.2 |  | 5.0 |  | 5.0 |  |
| 2 | No |  | 0.46 |  |  | 4.2 |  | 5.0 |  | 5.0 |  |
| 3 | No |  | 0.75 |  |  | 3.7 |  | 5.0 |  | 5.0 |  |
| 4 | No |  | 0.67 |  |  | 3.2 |  | 4.9 |  | 5.0 |  |
| 5 | Yes |  | 0.39 |  |  | 2.5 |  | 4.7 |  | 5.0 |  |
| 6 | No |  | 0.67 |  |  | 2.8 |  | 4.8 |  | 5.0 |  |
|  |  |  |  |  |  |  |  |  |  |  |  |
|  |  |  | P-value | LSD |  | P-value | LSD | P-value | LSD | P-value | LSD |
|  |  |  | 0.14 | 0.325 |  | 0.10 | 1.18 | 0.54 | 0.461 | >0.99 | * |

It should be noted that at d29 fresh litter was added to the pens. Despite this, litter score was equal and highest (= worst) at d35 for all treatments.

For footpad dermatitis, the scoring classes were: 0, no evidence of footpad dermatitis; 1, light footpad dermatitis; 2, severe foot pad dermatitis.

Litter was scored as: 1, loose; 2, most loose and a little sticky; 3, pressed together; 4, pressed together and wet; and as 5, wet and pappy.
